# The anticipatory and consummatory interpersonal pleasure scale: Applicability to Chinese OCD patients

**DOI:** 10.3389/fpsyg.2023.1074180

**Published:** 2023-02-03

**Authors:** Jingjie Lu, Xiang Wang, Qian Liu, Quanhao Yu, Jie Fan, Xiongzhao Zhu

**Affiliations:** ^1^Medical Psychological Center, the Second Xiangya Hospital, Central South University, Changsha, Hunan, China; ^2^Medical Psychological Institute of Central South University, Changsha, Hunan, China; ^3^National Clinical Research Center for Mental Disorders, Changsha, Hunan, China

**Keywords:** anticipatory and consummatory interpersonal pleasure scale, social anhedonia, obsessive–compulsive disorder, psychometric properties, measurement invariance

## Abstract

As a transdiagnostic symptom, social anhedonia has gained increasing attention. Evidence suggests that obsessive–compulsive disorder (OCD) patients demonstrate social anhedonia. This study examined the psychometric properties of the Anticipatory and Consummatory Interpersonal Pleasure Scale (ACIPS) in an undergraduate sample and Chinese OCD patients. Furthermore, we explored the relationship between clinical symptoms and ACIPS scores. This study involved 3,306 undergraduate students and 293 patients with OCD. Internal consistency and convergent validity of ACIPS were examined. Confirmatory factor analysis (CFA) was applied to determine the best-fitting of potential factor models, and multi-group CFA was used to examine measurement invariance across genders and samples. Additionally, hierarchical linear regression was conducted in order to investigate the relationship between clinical symptoms and ACIPS scores in patients suffering from OCD. ACIPS showed acceptable internal consistency in undergraduate and OCD samples (Cronbach’s α = 0.93 and 0.89, respectively). In both samples, the four-factor structure had the best fit index. Scalar invariance was established across undergraduate and OCD samples, while residual invariance was established across genders. In both samples, the ACIPS was significantly correlated with the Revised Social Anhedonia Scale and Beck Depression Inventory. Depression and the severity of obsessive thoughts significantly and negatively correlated with the ACIPS score in OCD patients (*p* < 0.05). In conclusion, ACIPS is a reliable, effective, simple, and convenient tool for the assessment of social anhedonia. Depression and obsessive thoughts contribute to social anhedonia in OCD patients.

## 1. Introduction

Anhedonia refers to a loss of interest in life or a diminished capacity to experience pleasure (Rizvi et al., 2016). It includes a diverse array of deficits in hedonic function and is a symptom associated with a wide range of psychiatric disorders (Loas, 1996). According to the content of pleasurable stimulus and its effects, anhedonia can be divided into physical and social anhedonia (Chapman et al., 1976). Social anhedonia is defined as a deficiency in pleasure from social contact, reduced reward experience from social stimuli and/or reduced motivation, and disinterest in pursuing relationships (Gooding and Pflum, 2022). In recent years, social anhedonia has been increasingly considered as a transdiagnostic symptom (Barkus and Badcock, 2019). Elevated rates of self-reported social anhedonia often associated with several disorders. For example, social anhedonia is a cardinal feature of schizophrenia-spectrum disorders (Pflum and Gooding, 2019). It is also prevalent among individuals with depression (Enneking et al., 2019), autism spectrum disorder (Gadow and Garman, 2020), post-traumatic stress disorder (Nawijn et al., 2015), and eating disorders (Tchanturia et al., 2012). Recently, accumulating evidence indicates that social anhedonia as a transdiagnostic process may also be an inherent feature of obsessive–compulsive disorder (OCD; Abramovitch et al., 2014, Xia et al., 2019, Grassi et al., 2020).

OCD is a well-known, burdensome psychiatric illness, and anhedonia may be considered an endophenotype candidate of OCD (Xu et al., 2020). It has been reported that about 1/3 OCD patients have demonstrated clinically significant anhedonia (Abramovitch et al., 2014). Evidence further suggested that social anhedonia was closely related to some obsessive–compulsive symptoms or disorders, such as hoarding(Chen et al., 2022) and Tourette syndrome (Eddy, 2018). A previous study provided neurobiological proof that substantiates the existence of the social anhedonia phenotype in OCD (Xia et al., 2019). Higher levels of social anhedonia are associated with lower levels of social support and social functioning (Blanchard et al., 2011), and adaptive social functioning is essential to recovery from mental illness (Barkus and Badcock, 2019). As OCD patients experience a deteriorated quality of life, especially on the aspect of social function (Macy et al., 2013), it may make it harder for them to experience pleasure in social interpersonal interaction. Therefore, it is meaningful to pay attention to social anhedonia in patients with OCD. As a prerequisite, it is necessary to define a standard and effective instrument for measurement of social anhedonia in OCD patients.

Several measures exist for measuring anhedonia, though only a few are specifically designed for measuring social anhedonia. One of the most commonly used instruments for social anhedonia is the revised Social Anhedonia Scale (RSAS; Eckblad et al., 1982). In addition, the Anticipatory and Consummatory Interpersonal Pleasure Scale (ACIPS) is a common method used to measure social anhedonia in patients and the general population (Gooding and Pflum, 2014a). Compared to the RSAS, the ACIPS measures social/interpersonal pleasure, which is an indirect measure of social anhedonia, reflecting more contemporary values, behaviors, and linguistic styles. Based on prior research, there exists a significant negative correlation between RSAS and ACIPS scores (Gooding and Pflum, 2014b). The ACIPS was specifically designed to identify individual differences in the ability to experience pleasure from social and interpersonal interactions, for example, sharing experiences with others, expressing feelings, and communicating feelings with others, either in person or remotely (Gooding et al., 2017). In addition to having more updated content, fewer items of the ACIPS could prevent patients with mental disorders from reliability and validity reduced due to overload with too many items, which may thereby reduce the credibility of results. ACIPS is easy to administer and is therefore time-saving. Besides the original adult version, the authors have developed a version for adolescents (Gooding et al., 2016b) and children, making the scale applicable to subjects of all ages.

Based on the advantages above, the ACIPS has been translated into numerous languages and is in wide use with many cross-cultural validations, including Mandarin Chinese, Spanish, French, and Korean (Chan et al., 2016; Gooding et al., 2016a; Chaix et al., 2017; Kim et al., 2022). The ACIPS demonstrated high internal consistency across studies (Cronbach’s α range from.86 to.95) in a wide range of college, community, and patient samples. The ACIPS has been administered to many patient groups, including patients with depressive disorder, schizophrenia-spectrum disorders, and autism, etc. (Ritsner et al., 2018; Han G. T., et al., 2019; Liang et al., 2020; Balasingam et al., 2023). The ACIPS has shown great validity (Gooding et al., 2014; Gooding and Pflum, 2014b; Gooding et al., 2015).These attributes made ACIPS a potentially helpful tool for elucidating how individual differences relate to risk for various forms of psychopathology linked to social relationship deficits.

The Chinese translation of ACIPS was validated psychometrically in 389 nonclinical participants, which indicates that it has four factors (Chan et al., 2016). Nonetheless, it is not known whether this scale is applicable to patients with OCD. We wondered whether the structure factors of social anhedonia in patients with OCD would be different from that in the undergraduate population. Furthermore, previous studies have found significant differences in ACIPS scores between male and female participants, with females reporting significantly greater levels of social and interpersonal pleasure (Gooding et al., 2015). However, to the best of our knowledge, no studies have been conducted on the measured invariance of ACIPS across genders, or across undergraduate and OCD samples. Measurement invariance refers to the comparability of scores obtained by people belonging to different groups on the same measurement instrument because the scores have a consistent meaning across the groups (Meredith, 1993). It may not be known whether these differences between groups are real without measurement invariance verification. Because the latent psychological structure may not be consistent across all groups. Simply put, there remains a need for measurement invariance testing of the ACIPS, which is vital for psychological research, particularly when comparing between groups.

This study aims to compare several different structural models previously reported, and to determine the most suitable model for the ACIPS in both undergraduate and OCD samples. Further, we assess the internal consistency reliability and convergent validity of ACIPS in OCD patients. Additionally, we use the best-fit factor structure to probe measurement invariance across OCD patients and undergraduate samples and across gender groups. Finally, we examine the presentation of ACIPS scores in patients with OCD and its relationship with clinical symptoms.

## 2. Methods

### 2.1. Participants

This study included an OCD sample and an undergraduate sample. A total of 305 (161 males,144 females) patients with OCD were recruited from the outpatient psychology department of the Second Xiangya Hospital. Two psychiatrists developed the diagnosis based on the structured clinical interview for axis I disorders (SCID-I) in the Diagnostic Statistical Manual of Mental Disorders, Fourth Edition (DSM-IV). Exclusion criteria: (1) Any Axis I psychiatric disorder comorbidity; (2) Neurological disorder comorbidity; (3) Physical disease-inducing knew psychiatric consequences (e.g., hypothyroidism, seizure disorder, brain injury); (4) History of psychoactive substance abuse or dependence.

We recruited 3,405 (1,125 males,2,280 females) undergraduate students from a university in Hunan Province of mainland China by convenient sampling method. Exclusion criteria were previous diagnosis of any psychiatric disorder, family history, and serious physical illness (based on self-report). All the participants were native Chinese speakers. Before they participated in the study, all subjects subscribed informed consent and the study was approved by Ethics Committee of the Second Xiangya Hospital of Central South University.

### 2.2. Instruments

Anticipatory and consummatory interpersonal pleasure scale (ACIPS) - The ACIPS contains 17 items that assess respondents’ ability to experience pleasure in interpersonal contexts (Gooding and Pflum, 2014a,b). A six-point Likert scale is used to rate items ranging from “very false” to “very true.” It has three versions: the adult version, adolescent version, and child version. In this study, we used adult version of the Chinese translation of the ACIPS (Chan et al., 2016).

Revised Social Anhedonia Scale (RSAS) – The RSAS consists of 40 items that assess social anhedonia, or the pleasure derived from social and interpersonal experiences, *via* statements that participants rate as true or false (Eckblad et al., 1982). A higher score indicates less pleasure from social interactions. The Chinese version of the RSAS has been shown to have good psychometric properties (Chan et al., 2012). In the current sample, Cronbach alpha coefficient was 0.889 for the undergraduate sample and 0.840 for the OCD sample.

Yale-Brown Obsessive–Compulsive Scale (Y-BOCS) - The Y-BOCS is the golden standard for assessing the severity of OCD symptoms (Goodman et al., 1989). The scale is comprised of 10 items, of which five assess the severity of obsessive thoughts, and five assess the severity of compulsive behavior. Each item was rated from 0 (no symptoms) to 4 (extreme symptoms). The Cronbach’s α was 0.846 in OCD sample in the current study.

Beck Depression Inventory (BDI) – The BDI is a self-report scale consisting of 21 items that assess depression severity (Beck et al., 1961). Each item was scored on a 4-point Likert scale (ranging from 0 to 3). BDI is the most commonly used scale to assess depression severity, and displays impressive reliability and validity. In the current study, Cronbach alpha coefficient was 0.929 for the undergraduate sample and 0.887 for the OCD sample.

State–Trait Anxiety Inventory (STAI) – The STAI contains 20 items to measure state anxiety(STAI-S, state-subscale) and 20 items to measure trait anxiety(STAI-T, trait-subscale; Spielberger et al., 1971). State anxiety items include: “I am tense; I am worried” and “I feel calm; I feel secure.” Trait anxiety items include: “I worry too much over something that really does not matter” and “I am content; I am a steady person.” All items are rated on a 4-point scale (e.g., from “Almost Never” to “Almost Always”). In this study, the Cronbach alpha coefficient of the STAI-S was 0.925 in the undergraduate sample. And the Cronbach alpha coefficient of the STAI-S and STAI-T was 0.936 and 0.897 in the OCD sample.

### 2.3. Data analysis

Confirmatory factor analysis (CFA) and measurement invariance analyses were analyzed in Mplus (version 8.3), while all others were in SPSS (version 26).

The structure validity of the ACIPS was examined using CFA with five different factor structures. These factor structures were identified by the number of factors and the country of the presenter, and are further described in the supplemental material. This study used a maximum likelihood with robust standards errors (MLR) method to perform the CFA, which is robust to departures from normality in the data distribution. Model fit was assessed using several indices, including Comparative Fit Index (CFI), Tucker–Lewis Index (TLI), Standardized, Root Mean Squared Residual (SRMR), and Root Mean Square Error of Approximation (RMSEA) with a 90% Confidence Interval (CI). When the following criteria are met, the model will be considered acceptable: CFI > 0.90, TLI > 0.90, SRMR <0.08 and RMSEA <0.08 (Hu and Bentler, 1999; Chen et al., 2008).

After verifying the best fitting model for the ACIPS, we conducted measurement equivalence tests across genders and across undergraduate participants and OCD patients by adding a series of increasingly strict constraints between groups. With increasing cross-group restrictions on parameters, four measurement invariance models were applied: (1) configural invariance, which tests factor structure invariance of factor latent variables across groups; (2) metric invariance, which tests factor loading invariance across groups; (3) scalar invariance, which tests intercept invariance across groups; and (4) residual invariance, which tests error variance invariance across groups. A non-significant △χ^2^ difference test (△χ^2^ test) was considered as evidence of invariance. However, the △χ^2^ test is sensitivity to sample size. As a result, CFI and RMSEA differences between increasingly constrained models, termed ΔCFI and ΔRMSEA, were used to evaluate model suitability for model confirmation. The criteria for acceptable invariance were ΔCFI ≤0.01, and ΔRMSEA ≤0.015 (Cheung and Rensvold, 1999).

We assigned each item to a scale and verified the scale’s internal consistency. Internal consistency was evaluated by calculating Cronbach’s α coefficient and mean inter-item correlation (MIC) values. Cronbach’s α values and MIC value greater than 0.7 and 0.15, respectively were considered acceptable. A Pearson correlation coefficient was used to assess the converging validity between the ACIPS total score and the RSAS and BDI scores.

Finally, regression analyses were used to analyze the relationship between social anhedonia and clinical variables in patients with OCD.

## 3. Results

### 3.1. Descriptive statistics

Ultimately, 12 patients with OCD and 99 undergraduate students were excluded from the analysis because more than two items were missing. The OCD sample (N = 293) was composed of 138 females (47.1%) and 155 males (52.9%), with a mean age of 23.51 years. The undergraduate sample (N = 3,306) included 2,216 females (67.0%) and 1,090 males (33.0%) with a mean age of 19.13 years.

As shown in Table 1, the mean age of the OCD sample was significantly higher than that of the undergraduate sample (*t* = 10.498; *p* < 0.01), and the proportion of females in the OCD sample was significantly lower than that of the undergraduate sample (*χ^2^* = 47.252; *p* < 0.01). However, the number of years of education was not significantly different between the two samples (*t* = 0.698; *p* < 0.486). In terms of clinical characteristics, RSAS, BDI, and STAI-S in the OCD group was significantly higher than in the undergraduate group (*p* < 0.01).

**Table 1:** Demographic and clinical characteristics of OCD patients and undergraduate students.

	OCD (*n* = 293)	Undergraduate (*n* = 3,306)	*t/χ^2^*	*p*
Age (in years)	23.51 (7.14)	19.13 (0.74)	10.498	<0.001
Gender (female, %)	138 (47.10)	2,216 (67.03)	47.252	<0.001
Education (in years)	13.82 (2.70)	13.71 (0.56)	0.698	0.486
RSAS	15.84 (6.93)	11.13 (6.14)	11.036	<0.001
BDI	18.13 (10.01)	4.43 (6.85)	22.409	<0.001
STAI-S	52.57 (12.76)	36.52 (10.15)	20.527	<0.001
STAI-T	55.85 (10.13)			
Y-BOCS-O	10.58 (3.60)			
Y-BOCS-C	8.74 (4.53)			
Duration (in months)	44.58 (55.85)			

1, Means with standard deviations in parentheses. 2, Abbreviations: OCD, obsessive–compulsive disorder; RSAS, Revised Social Anhedonia Scale; BDI, Beck Depression Inventory; STAI-S/T, Spielberger State–Trait Anxiety Inventory-State Form /Trait Form, Y-BOCS, The Yale-Brown Obsessive–Compulsive Scale; Y-BOCS-C, compulsive-subscale of Y-BOCS; Y-BOCS-O, obsessive-subscale of Y-BOCS. 3, t/χ^2^: Categorical data of gender was tested using chi squared tests (indicated by χ^2^); other variables were tested by two-sample t-test (indicated by t).

Regarding ACIPS scores, in the OCD sample, the mean (±standard deviation) ACIPS score was 67.05 ± 13.94. There was no significant difference between males (N = 155, 66.83 ± 14.09) and females (N = 138, 67.29 ± 13.82; *t* = −0.280, *p* = 0.78). In the undergraduate sample, the mean (±standard deviation) ACIPS score was 76.56 ± 12.96. Males (N = 1,090, 74.10 ± 13.35) had a lower mean score of the ACIPS than females (N = 2,216, 77.75 ± 12.59; *t* = −7.390, *p* < 0.001). The mean ACIPS score was higher in undergraduate students than in patients with OCD (*t* = −11.958; *p* < 0.01).

### 3.2. Factor structure of ACIPS

The fit index values obtained for each of the five different structure models we compared were reported in Table 2. The Chinese four-factor structure of ACIPS provided the best fit for the data in both undergraduate sample (CFI = 0.929, TLI = 0.915, SRMR = 0.037, RMSEA = 0.057) and OCD sample (CFI = 0.917, TLI = 0.900, SRMR = 0.049, RMSEA = 0.058).

**Table 2:** Fit of factorial models of the ACIPS in undergraduate sample and OCD sample.

Model	χ^2^ (df), *p*	CFI	TLI	SRMR	RMSEA (90%CI)
Undergraduate sample (*n* = 3,306)					
Model 1	1582.836 (118), *p* < 0.001	0.914	0.901	0.039	0.061 (0.059–0.064)
Model 2	1597.126 (116), *p* < 0.001	0.913	0.898	0.039	0.062 (0.059–0.065)
Model 3	1433.118 (116), *p* < 0.001	0.923	0.909	0.037	0.059 (0.056–0.061)
Model 4*	–	–	–	–	–
**Model 5**	**1318.086 (113), *p* < 0.001**	**0.929**	**0.915**	**0.037**	**0.057 (0.054–0.060)**
OCD sample (*n* = 293)					
Model 1	244.115 (118), *p* < 0.001	0.906	0.891	0.050	0.060 (0.050–0.071)
Model 2	244.935 (116), *p* < 0.001	0.904	0.887	0.050	0.062 (0.051–0.072)
Model 3	231.185 (116), *p* < 0.001	0.914	0.899	0.048	0.058 (0.047–0.069)
Model 4	258.610 (113), *p* < 0.001	0.891	0.869	0.050	0.066 (0.056–0.077)
**Model 5**	**224.737 (113), *p* < 0.001**	**0.917**	**0.900**	**0.049**	**0.058 (0.047–0.069)**

Model 1, two-factor structure; Model 2, original three-factor structure; Model 3, Korean three-factor structure; Model 4, Spanish three-factor structure; Model 5, Chinese four-factor structure; χ^2^, chi-square; df, degree of freedom; CFI, comparative fit index; TLI, Tucker-Lewis index; RMSEA, root-mean-square error of approximation; SRMR, standardized root mean square residual; CI, confidence interval. *Model 4 does not converge in undergraduate sample.

### 3.3. Measurement invariance

The Chinese four-factor structure model had adequate fit among all subgroups (undergraduate sample, OCD sample, male group, and female group). As reported in Table 3, We examined the configural, metric, scalar and residual invariance between undergraduate and OCD samples sequentially. The indices all satisfy the recommended requirements that mentioned before (CFI = 0.929, TLI = 0.914, SRMR = 0.038, RMSEA = 0.060), indicating that the configural invariance was accepted. Further the metric invariance model was compared with the configural invariance model, ΔCFI was equal to−0.002, indicating that the metric invariance was accepted. Moreover, the scalar invariance model was compared with the metric invariance model, ΔCFI was equal to-0.004 indicating that the scalar invariance was accepted. Finally, the residual invariance model was compared with the scalar invariance model, and there was a significant change in the model fit (ΔCFI = -0.029), indicating that the residual variance differed across groups. In other words, residual invariance was not supported.

**Table 3:** Measurement invariance model fitting indices and comparison.

Model	χ^2^(df), *p*	CFI	TLI	SRMR	RMSEA	Model comparison	∆CFI	∆TLI	∆RMSEA	Decision
Invariance across undergraduate and OCD samples										
Model 1	1682.865 (226), *p* < 0.001	0.929	0.914	0.038	0.060	–	–	–	–	–
Model 2	1729.928 (239), *p* < 0.001	0.927	0.917	0.041	0.059	2 vs. 1	−0.002	0.003	−0.001	Accept
Model 3	1835.970 (252), *p* < 0.001	0.923	0.917	0.043	0.059	3 vs. 2	−0.004	0.000	0.000	Accept
Model 4	2450.604 (269), *p* < 0.001	0.894	0.892	0.060	0.066	4 vs. 3	−0.029	−0.025	0.007	Reject
Invariance across gender groups										
Model 1	1600.173 (226), *p* < 0.001	0.925	0.910	0.039	0.058	–	–	–	–	–
Model 2	1645.456 (239), *p* < 0.001	0.923	0.913	0.045	0.057	2 vs. 1	−0.002	0.003	−0.001	Accept
Model 3	1812.277 (252), *p* < 0.001	0.915	0.908	0.049	0.059	3 vs. 2	−0.008	−0.005	0.002	Accept
Model 4	1846.272 (269), *p* < 0.001	0.914	0.913	0.055	0.057	4 vs. 3	−0.001	0.005	−0.002	Accept

Model 1 = configural invariance; Model 2 = metric invariance; Model 3 = scalar invariance; Model 4 = residual invariance; χ^2^, chi-square; df, degree of freedom; CFI, comparative fit index; TLI, Tucker-Lewis index; RMSEA, root-mean-square error of approximation; SRMR, standardized root mean square residual; ∆, change in the parameter.

Likewise, we also tested four hierarchically constrained models across gender groups. In the configural invariance test, various parameters were allowed to be freely estimated, and the indices all satisfy the recommended requirements that mentioned before (CFI = 0.925, TLI = 0.910, SRMR = 0.039, RMSEA = 0.058). Based on the configural invariance, we conduct next three invariance tests successively. The changes of CFI, TLI and RMSEA (∆CFI < 0.010, ∆TLI < 0.010, ∆RMSEA <0.015) supported metric, scalar and residual invariance (see Table 3). That is to say, the measurement invariance across gender met the acceptance criteria through residual invariance.

Hence, the ACIPS was confirmed to have equal factor structure, metrics, and intercepts between women and men, as well as between undergraduate and OCD samples.

### 3.4. Reliability and convergent validity

In undergraduate sample, we obtained a Cronbach’s α of 0.93, a MIC of 0.44 for the whole scale. The Cronbach’s α for four factors: Friendship, Family and Intimacy-related Relationships, General Social Interactions, and Casual Interactions/Conversations were 0.87, 0.69, 0.80 and 0.57, and MIC were 0.53, 0.40, 0.46 and 0.41. Similarly, for OCD sample, we obtained a Cronbach’s α of 0.894, a MIC of 0.34 for the whole scale. And the Cronbach’s α of Factor 1-Factor 4 were from 0.53 to 0.79, and MIC was from 0.32 to 0.38. ACIPS total scores were significantly and negatively correlated with RSAS scores (undergraduate sample: *r* = −0.537; OCD sample: *r* = −0.610) and BDI scores (undergraduate sample: *r* = −0.241; OCD sample: *r* = −0.247). These results suggested the ACIPS had good convergent validity in both samples.

### 3.5. The contribution of clinical symptoms to ACIPS score in OCD patients

The second aim of this study was to examine the contribution of clinical symptoms to social anhedonia in patients with OCD. A hierarchical linear regression analysis was conducted to investigate depression level (BDI scores), state or trait anxiety level (STAI-S and STAI-T scores), and severity of obsessive and compulsive symptoms (Y-BOCS-O and Y-BOCS-C scores) as predictors for patients with OCD after controlling for age, gender, and education years. The results showed that depression (*β* = −0.168, *p* < 0.05) and the severity of obsessive thoughts (*β* = −0.133, *p* < 0.05) significantly and negatively correlated the ACIPS score (see Table 4).

**Table 4:** Predictors of ACIPS scores in patient with OCD.

	Predictors	B	β	t	F	ΔR^2^
Step 1	Age	0.059	0.030	0.454	0.594	0.006
	Gender	0.008	0.000	0.005		
	Education	−0.046	−0.009	−0.146		
Step 2	**BDI**	**−0.272**	**−0.190**	−2.387*	3.168**	0.092
	STAI-S	−0.141	−0.126	−1.452		
	STAI-T	0.190	0.137	1.441		
	**Y-BOCS-O**	**−0.606**	**−0.155**	−2.266*		
	Y-BOCS-C	−0.035	−0.015	−0.252		
	Duration	0.020	0.078	1.155		

OCD, obsessive–compulsive disorder; BDI, Beck Depression Inventory; STAI-S/T, Spielberger State–Trait Anxiety Inventory-State Form /Trait Form, Y-BOCS, The Yale-Brown Obsessive–Compulsive Scale; Y-BOCS-C, compulsive-subscale of Y-BOCS; Y-BOCS-O, obsessive-subscale of Y-BOCS. * *p*< 0.05, ** *p*< 0.01.

## 4. Discussion

The purpose of this study was to examine the psychometric characteristics of the ACIPS in Chinese OCD patients. The study also analyzed the relationship between the severity levels of various clinical symptoms of OCD and the ACIPS score. The results of CFA showed that the four-factor model of ACIPS was optimal, with good internal consistency reliability and convergent validity. The study therefore confirmed that the scale is effective in measuring social anhedonia with measurement invariance across genders, as well as across OCD patients and undergraduate populations. The ACIPS scores were significantly higher among undergraduates than in the patients with OCD. In the undergraduate sample, females experienced significantly more social pleasure than males, while no gender difference was found in the OCD sample. In addition, the study indicates that depression and the severity of obsessive thoughts was significantly correlated with the level of social pleasure deficit in OCD patients.

Previous studies probing the psychometric properties of the ACIPS in different languages have reported inconsistencies in the scale’s factor structure. The ACIPS was originally designed to distinguish between anticipatory and consummatory pleasure, forming a two-factor structure, but subsequent analyses were not able to confirm its validity. Instead, evidence suggests that either a three-or four-factor model is more appropriate (Gooding and Pflum, 2014a, 2016; Chan et al., 2016; Gooding et al., 2016a; Chaix et al., 2017; Kim et al., 2022). Lately, researchers validated the structure of the ACIPS in a mixed clinical sample (including 294 patients with schizophrenia, bipolar disorder, or major depressive disorder), replicated the four-factor structure of the ACIPS in a Chinese setting, and showed good discrimination validity (Liang et al., 2020). Consistent with that, in this study, the results of a confirmatory factor analysis indicate that a four-factor solution provides an appropriate fit in the Chinese OCD patient sample. This includes Friendship, Family and Intimacy-Related Relationships, Social Interactions, and Casual Interactions/Conversations.

It is necessary to ensure measurement invariance before making a comparison among group means, in order to determine whether a construct has the same meaning across groups (Han K. et al., 2019). In this study, scalar invariance was established across undergraduate and OCD samples, and residual invariance was established across genders, which suggests that social anhedonia manifests similarly across groups. The measurement invariance across groups allows us to interpret the measurement data of social anhedonia in a meaningful way. The level of interpersonal pleasure in OCD patients was significantly lower than in the undergraduate sample, implying general social anhedonia in OCD patients, which is consistent with previous studies (Xia et al., 2019). Interacting with others involves a great deal of social cognition, i.e., cognitions about the thoughts, feelings, and behaviors of others. Recently, a meta-analysis showed that OCD is associated with medium-sized deficits in the theory of mind and cognitive empathy (Bora, 2022). Impairments in social cognition render social interactions more difficult and less enjoyable. In this way, although social functioning domains are not primary hedonic deficits, they do contribute to social anhedonia (Gooding et al., 2016a). In the undergraduate sample, we observed gender differences in that females reported higher levels of social and interpersonal pleasure than males, in full agreement with previous studies (Gooding et al., 2015). In contrast, no gender differences were found in the OCD patients.

In terms of reliability, the ACIPS has good internal consistency in both the undergraduate sample and the OCD patients. The results of convergent validity indicated that individuals with lower interpersonal pleasure as assessed by ACIPS had a high level of social anhedonia measured by the RSAS, which is consistent with the previous studies (Gooding and Pflum, 2014b). The ACIPS was inversely associated with the BDI, consistent with prior findings in Spanish and Korean samples (Gooding et al., 2016a; Kim et al., 2022).

The second aim of the current study was to explore the relationship between clinical symptoms and ACIPS scores in patients with OCD. The result demonstrated that depression and the severity of obsessive thoughts significantly and negatively correlated with the ACIPS score in patients with OCD. A study found that major depression appears to be associated with state-related social anhedonia (Blanchard et al., 2001). In patients with schizophrenia and bipolar disorder, researchers have also found that higher social anhedonia scores were primarily predicted by greater depression scores (Bodapati et al., 2019). The relationship between depression levels and social pleasure deficits appears to have a cross-diagnostic trend. As for the effects of obsessive thoughts, according to the earlier discussion, people suffering from OCD tend to have social anhedonia. Research has demonstrated that the clinically severe subgroups of OCD reported the highest impairment ratings in relationships and social functioning domains (Ruscio et al., 2010). One possible reason is that individuals with OCD already have impaired emotional awareness and perception (Kang et al., 2012), and individuals with high levels of obsessive–compulsive thinking are more perfectionistic, making it more difficult to have pleasant experiences in interpersonal interactions. In addition, patients with OCD often struggle with hostile and suspicious thoughts, and studies have shown a positive correlation between hostility and the severity of OCD (Tellawi et al., 2016). This may make them more cautious about interacting with others and less likely to experience social pleasure. For patients with OCD without comorbid other psychiatry disorders, hostility is often implied only in obsessive thoughts rather than directly in behavior.

The present study had some advantages in that it was the first study to compare all the factor structures of available versions of ACIPS in both undergraduate and OCD samples. In addition, for the first time, we explored and validated the measurement invariance of ACIPS across gender and groups, meeting the criteria to allow intergroup comparisons. At the same time, there were some limitations of this study. For example, no specific question is set to prevent random responses. In addition, the sample included in the survey is still relatively simple. To examine measurement invariance on more epidemiological dimensions, future studies should extend the sampling range and sample size of subjects. In addition, the measurement invariance of ACIPS across time remains to be verified.

## 5. Conclusion

In summary, the ACIPS was demonstrated to have good reliability and validity in an undergraduate sample and a sample of OCD patients. In both samples, the four-factor structure had the best fit index. Scalar invariance was observed between undergraduate and OCD samples, while residual invariance was established across genders. Furthermore, social anhedonia in OCD patients is associated with depression and obsessive thoughts. In conclusion, the ACIPS is a reliable, effective, simple, and convenient tool for assessing and screening for social anhedonia.

## Data availability statement

The raw data supporting the conclusions of this article will be made available by the authors, without undue reservation.

## Ethics statement

The studies involving human participants were reviewed and approved by Ethics Committee of the Second Xiangya Hospital, Central South University. Written informed consent to participate in this study was provided by the participants’ legal guardian/next of kin.

## Author contributions

XZ conceived and designed the study. The data analysis, results interpretation, and original manuscript writing were done by JL. XW, XZ, QL, and QY reviewed the original manuscript. QL, XW, JL, and QY collected the research data. JF and XZ supervised this work. All authors contributed to the article and approved the submitted version.

## Funding

This work was supported by grants from the National Natural Science Foundation of China (XZ, grant number 82171532).

## Conflict of interest

The authors declare that the research was conducted in the absence of any commercial or financial relationships that could be construed as a potential conflict of interest.

## Publisher’s note

All claims expressed in this article are solely those of the authors and do not necessarily represent those of their affiliated organizations, or those of the publisher, the editors and the reviewers. Any product that may be evaluated in this article, or claim that may be made by its manufacturer, is not guaranteed or endorsed by the publisher.
