# Supplementary material for: The anticipatory and consummatory interpersonal pleasure scale: Applicability to Chinese OCD patients
Source: Front Psychol. 2023 Feb 3;14:1074180. doi: 10.3389/fpsyg.2023.1074180 (PMC9936859; doi:10.3389/fpsyg.2023.1074180)
Supplement: Supplementary file 1 [file Table_1.DOCX]

Supplementary Material

**Supplementary Table 1(S1)**: Five different factor structures of different versions of ACIPS.

| Factor Model | F1 | F2 | F3 | F4 |
| --- | --- | --- | --- | --- |
| Model 1 | 1,3,7,8,10,14,15 | 2,4,5,6,9,11,12,13,16,17 |  |  |
| Model 2 | 2,3,6,7,9,10,14,17 | 1,4,11,13 | 5,8,12,15,16 |  |
| Model 3 | 4,5,6,7,8,10,11,12,15 | 1,9,13,14,16,17 | 2,3 |  |
| Model 4 | 2,6,7,8,9,14 | 1,3,4,5,10,11,12,13,17 | 5,8,12,15,16 |  |
| Model 5 | 8,9,13,14,15,17 | 3,5,10,12 | 1,2,6,7,11 | 4,6 |

*NOTE: Model 1 = two-factor structure; Model 2 = original three-factor structure; Model 3 = Korean t**hree-factor structure; Model 4 = Spanish three-factor structure; Model 5 = Chinese four-factor structure*

** The French version of ACIPS is consistent with the original three-factor structure.*
